# “Ebinyo”—The Practice of Infant Oral Mutilation in Uganda

**DOI:** 10.3389/fpubh.2017.00167

**Published:** 2017-07-17

**Authors:** Margaret N. Wandera, Betsy Kasumba

**Affiliations:** ^1^Uganda Dental Association, Kampala, Uganda; ^2^Makerere University, Kampala, Uganda

**Keywords:** infant oral mutilation, ebinyo, traditional healers, Uganda, canines

## Abstract

Infant oral mutilation (IOM) is a traditional method of extracting un-erupted teeth practiced in several Sub-Saharan African countries including Uganda. This practice is referred to as “ebinyo” by Bantu-speaking Ethnic groups, though it has several terms depending on cultural group and researcher. The un-erupted tooth is gouged out as a cure for medical symptoms in infants that include high fevers and diarrhea. The spreading of IOM practice in African populations is blamed on poor health literacy with regard to the common childhood illnesses. One study in Uganda revealed that adverse cases following IOM seen in the hospital peaked in tandem with the malaria and diarrheal disease cases. This paper is a review of the practice with a particular focus on Uganda as presented in literature compiled from PubMed, Dentaid, Google Scholar, Local Uganda sources, and the authors’ observations. The paper explains reason for the persistence of the practice, and to further inform on IOM to health practitioners who were previously unaware of the practice.

## Introduction

Traditional methods of treating illnesses are still practiced in several parts of Africa (1–3). These traditional methods may not be scientifically explainable, yet societies continue to apply them as a means to prevent and treat diseases. World Health Organization reports that 80% of African populations use traditional medicine for cultural and economic reasons as their primary source of care (3).

Traditional methods of treatment may be injurious as has been observed by several authors on a practice referred to as infant oral mutilation (IOM). In the literature, authors also refer to IOM by other terms that include *tooth extirpation, germectomy, deciduous canine buds enucleation, nylon teeth*, and *false teeth* (4–7). The most common term for IOM practice is Ebinyo which is derived from the Bantu- languages and loosely translates to “false teeth.”

Infant oral mutilation is where un-erupted teeth, usually in the position of canines, are gouged out by a non-formally trained person. The raised areas on the infants gum are identified and then using a sharp instrument the soft un-mineralized tooth is extracted as the “offending worm.” The range of rudimentary that may be used include bicycle spokes, hot needles, pointed knives, nails, and other sharp objects (5, 6, 8–10). The procedure is carried out in the belief that it will prevent or treat symptoms such as fevers or diarrhea seen in an infant (5, 7–10).

Gollings (active document) reports that the earliest literature report on the practice was found in tribes of the Nilotic Sudan in 1932. The practice is now reported to have spread to several Sub-Saharan Africa countries that include Uganda, Chad, Sudan, Ethiopia, Somalia, DR Congo, Kenya, Tanzania, Rwanda, and Burundi. Studies also report individuals migrating from these African nations may continue this practice in Europe, Australia, and the Americas (11–14).

## Method

This paper is a review of IOM in Uganda. It has been compiled from PubMed, Dentaid, Google Searches, Local Uganda sources, and supplemented by anecdotal evidence from the authors. The search terms were as follows: Infant oral Mutilation, Ebinyo, Ebino, Canine extirpation, and enucleation. The authors reviewed articles, media presentations, and reports.

## History of IOM in Uganda

Uganda is a landlocked country located in the Eastern part of Africa. Uganda is bordered by South Sudan to the North, Kenya to the East, DR Congo to the West, and Rwanda and Tanzania to the South. Uganda gained its independence from Britain in 1962. There was a military coup in 1971, followed by a period of instability in the country up until 1986; as a result, this period has scarce documented research.

The current population of Uganda is approximately 40 million. Uganda has a rapid population growth, reported as increasing from 9.5 million in 1969 to 35 million in 2014 (15). Only 25% of the population lives in urban areas. Uganda has several ethnic groups, each with their own language, customs, and traditional practices (16). These ethnic groups can be grouped into Bantu speaking and non-Bantu speaking, with the former living mainly in the Southern regions while the latter living in the Northern regions.

The first mention of IOM in Uganda is in a study carried out in 1969 by Pindborg (17). They reported that 16.1% of the children of the Acholi tribe of Northern Uganda had missing canines due to IOM. The dental mutilation gave credence to the existing myth to extract teeth as a remedy to childhood fevers.

In 1971, Halestrap who had observed some dental anomalies in Uganda populations caused by customary and superstitious practices assumed they were on the decrease as modern health practices were being adopted. He then proceeded to document the practices so as to keep a written record under the assumption that the practices would be disused in later years (18). This author clearly described the regional distribution of the different traditional practices in Ugandan cultural groups, stating that the “deciduous teeth enucleation” was only practiced in the Northern region of the Uganda and not in any other parts. This is in concordance with the findings of Pindborg as a practice of the Northern region. When the country was more stable, in 1989, the Uganda Ministry of Health carried out a survey and reported 95% of a focus group they studied in a Southern district of Uganda had heard of Ebinyo, thereby indicating a spread (19).

## Trends of IOM Practice in Uganda

Infant oral mutilation is currently reported all over Uganda. In 40 years, IOM prevalence is now reported by Tiromwe et al. (20) to have almost tripled to over 50% in the Northern district that Pindborg studied (17). Furthermore, the tribe of the Baganda that had no traditional practices interfering with their normal dentition in 1971, in more recent studies is implicated as having introduced IOM to the South Western regions (9, 10). Literature further states that elder persons are more likely to report IOM as a new condition, while the younger people believed the practice always existed (5, 9, 10). Similarly, the authors (Betsy Kasumba and Margaret N. Wandera) have observed dental consultations at conventional clinics are more likely from grandmothers rather than mothers before taking a child for “Ebinyo” treatment. IOM has been found to be done more in rural, than urban children, and more likely to be done on children who were under the care of a caretaker than a parent (20). However, the levels in urban areas are considerably high, as reported in a study of children attending child clinic in the Capital city Kampala in 2007 where 24% who had undergone IOM.

## Impacts of IOM

The adverse impacts of this procedure may be categorized into the immediate and the long term. Since the fever or diarrhea symptoms of the infant do not get the appropriate treatment, there is the likelihood of the pre-existing illness to worsen. Additionally, the non-sterile invasive method used to gouge the tooth out may result in bleeding and infection. These may be so severe to cause anemia, septicemia, osteomyelitis, or meningitis (8, 20–23). A study of hospital admissions in Northern Uganda observed that children who had undergone IOM were among the 10 most common hospital admissions and had the third highest case fatality rate (CFR = 21%) (24).

The long-term impacts are observed especially in the dentition and include malformation, non-eruption, hypoplasia, dysplasia, missing teeth, displacement and impaction, compound odontoma, and orthodontic complications (7, 11, 17, 21). The teeth most commonly affected are the mandibular canines. A study of 14-year olds in the city of Kampala found that in the mandible, missing canines were as common as missing first molars. The occurrence of missing canines could be explained as result of IOM and had impact on the children occlusal status (25).

## Attitudes of Uganda Populations to IOM

In Uganda, IOM is carried out commonly by traditional healers, though other respected members of the community may conduct the procedure. The literature mentions family members, traditional midwives, school teachers, and even local priests conducting IOM (5, 20, 22). Traditional healers remain widespread in Uganda as with most of Africa, especially in the rural areas where the populations rely greatly on their services. In a study of traditional healers, 40% had no formal education, whereas 46.6% had only primary school education (26). Traditional healers take up their role as a cultural heritage. Ellis and Arubaku (23) state that families initially consult a traditional healer before hospital, and even while at hospital may continue the dialog. In the National Oral Health Plan, when participants were asked about *Ebinyo*, more than 50% stated that the best treatment is by a traditional healer (19). A later study conducted in a Kampala clinic in 2007, guardians reported traditional healers were responsible for 55% of IOM observed (*MW*). The authors conclude that this reflected the poor health literacy of the studied Uganda population. In a study by Nuwaha et al. (27) in a western part of Uganda, it is reported that socioeconomic conditions do not influence IOM as a preferred choice of treatment. In neighboring Tanzania, IOM was outlawed in 1980s, but as of 1990s, it was still occurring in areas that have poor access to health services (28). Therefore, IOM persistence and spread may be due to poor health literacy and limited access to health services in these populations.

This continued inhumane practice of IOM is conducted at an age where the antibodies protection passed on to a child during pregnancy and from breast milk is decreasing. The child, thus, becomes susceptible to various infections. These infections present with symptoms of fevers, diarrhea, and vomiting that IOM is performed to treat. Notably, a high proportion of morbidity and mortality in under 5-year olds in Sub-Saharan Africa is from these infections (27, 29). The prevailing mismanagement of the infections, such as IOM in the Uganda population, thus presents as a contributory factor to the health burden of children (2).

## Conclusion

Infant oral mutilation should be eradicated. The interventions ought to involve traditional healers and offer improved access to primary health care, especially in rural areas. The majority of the studies in literature focus on the dental impacts of IOM with minimal reporting on the reasons for delay seeking proper care from conventional health services. Health professionals in particular pediatricians should be informed and liaise with dental practitioners to develop strategies to eliminate this practice. Further research into the conditions that are promoting such beliefs should be explored.

## Author Contributions

MW and BK contributed to the concept of the manuscript. Furthermore, they worked together to develop the design and select the content. Both authors agreed to the final version and approved it ready for submission.

## Conflict of Interest Statement

The authors declare that the research was conducted in the absence of any commercial or financial relationships that could be construed as a potential conflict of interest.
